# Developing and implementing new clinical academic partnerships: the King’s Maudsley partnership

**DOI:** 10.1192/j.eurpsy.2026.10276

**Published:** 2026-07-31

**Authors:** P. Shaw

**Affiliations:** Child and Adolescent Psychiatry, King’s Maudsley Partnership, London, United Kingdom

## Abstract

**Abstract:**

The King’s Maudsley Partnership at the Pears Maudsley Centre is the UK’s first fully integrated clinical and research facility dedicated to children and young people’s mental health. The partnership brings together clinical services (South London and Maudsley NHS Trust), academics (Child Psychiatry Department at the Institute of Psychiatry, Psychology and Neuroscience) and the Maudsley Charity. This session will explore how the KMP team aim to integrate research and practice, transforming discovery science, accelerating early clinical translation and delivering impactful implementation.

In this first talk, I will review some of the barriers to progress in child and adolescent psychiatry, highlighting how many stem from a lack of joint working between key partners. I will then consider ways forward: how the Partnership and its new home at the Pears Maudsley Centre might overcome some barriers. This could help clinicians accelerate the early translation of new findings, and academics conducting research that is embedded in clinically vital questions.

**Disclosure of Interest:**

None Declared

